# Fatty acid amide hydrolase inhibitors confer anti-invasive and antimetastatic effects on lung cancer cells

**DOI:** 10.18632/oncotarget.7592

**Published:** 2016-02-22

**Authors:** Katrin Winkler, Robert Ramer, Sophie Dithmer, Igor Ivanov, Jutta Merkord, Burkhard Hinz

**Affiliations:** ^1^ Institute of Toxicology and Pharmacology, Rostock University Medical Center, Rostock, Germany

**Keywords:** FAAH inhibitors, endocannabinoids, metastasis, invasion, human lung cancer cells

## Abstract

Inhibition of endocannabinoid degradation has been suggested as tool for activation of endogenous tumor defense. One of these strategies lies in blockade of fatty acid amide hydrolase (FAAH) which catalyzes the degradation of endocannabinoids (anandamide [AEA], 2-arachidonoylglycerol [2-AG]) and endocannabinoid-like substances (N-oleoylethanolamine [OEA], N-palmitoylethanolamine [PEA]). This study addressed the impact of two FAAH inhibitors (arachidonoyl serotonin [AA-5HT], URB597) on A549 lung cancer cell metastasis and invasion. LC-MS analyses revealed increased levels of FAAH substrates (AEA, 2-AG, OEA, PEA) in cells incubated with either FAAH inhibitor. In athymic nude mice FAAH inhibitors were shown to elicit a dose-dependent antimetastatic action yielding a 67% and 62% inhibition of metastatic lung nodules following repeated administration of 15 mg/kg AA-5HT and 5 mg/kg URB597, respectively. *In vitro*, a concentration-dependent anti-invasive action of either FAAH inhibitor was demonstrated, accompanied with upregulation of tissue inhibitor of matrix metalloproteinases-1 (TIMP-1). Using siRNA approaches, a causal link between the TIMP-1-upregulating and anti-invasive action of FAAH inhibitors was confirmed. Moreover, knockdown of FAAH by siRNA was shown to confer decreased cancer cell invasiveness and increased TIMP-1 expression. Inhibitor experiments point toward a role of CB_2_ and transient receptor potential vanilloid 1 in conferring anti-invasive effects of FAAH inhibitors and FAAH siRNA. Finally, antimetastatic and anti-invasive effects were confirmed for all FAAH substrates with AEA and OEA causing a TIMP-1-dependent anti-invasive action. Collectively, the present study provides first-time proof for an antimetastatic action of FAAH inhibitors. As mechanism of its anti-invasive properties an upregulation of TIMP-1 was identified.

## INTRODUCTION

Cannabinoids have been demonstrated to exert anticarcinogenic effects via multiple mechanisms. Within past years several studies have indicated that, apart from its well-known proapoptotic and antiproliferative properties, cannabinoids are potent anti-invasive and antimetastatic agents in different cancer models (for review see [1]).

Besides activating cannabinoid receptors by administering agonists exogenously, another tool for therapeutical intervention lies in the activation of these receptors by increasing the levels of endocannabinoids locally at pathological foci. This strategy has attracted substantial interest in recent years on the basis of findings showing increased endocannabinoid levels in certain cancer types [2, 3]. It was therefore hypothesized that the endocannabinoid system represents an endogenous tumor defense complex, i.e. cannabinoids produced following the onset of cancer may counteract neoplasia site-specifically (for review see [4]). Thus, selective inhibitors of endocannabinoid degradation may serve as useful and effective tool for activation of the endogenous tumor defense.

One of these strategies lies in inhibition of the enzyme fatty acid amide hydrolase (FAAH), a member of the serine hydrolase family of enzymes that was first identified as the principal catabolic enzyme of the endocannabinoid anandamide (N-arachidonoylethanolamine, AEA) [5]. FAAH is likewise the catabolic enzyme for other fatty acid amides, including N-oleoylethanolamine (OEA) and N-palmitoylethanolamine (PEA) [6]. 2-Arachidonoylglycerol (2-AG), the second major endocannabinoid, can be hydrolyzed by multiple enzymes, including FAAH and monoacylglycerol lipase (MAGL) with about 85% of brain 2-AG hydrolase activity ascribed to MAGL [7].

Several nonselective and selective inhibitors of FAAH have been described meanwhile, including URB597, a relatively selective, irreversible, carbamate-based inhibitor [8–10], and arachidonoyl serotonin (AA-5HT), an endogenous molecule first described as FAAH inhibitor [11]. AA-5HT has later been identified as antagonist at the transient receptor potential vanilloid 1 (TRPV1) thereby posessing analgesic properties [12]. In contrast to the unwanted side effects of conventional CB_1_ receptor agonists, FAAH inhibitors have been demonstrated to elicit no hypomotility [13] and cataleptic behavioral responses [14]. In addition, FAAH inhibitors have proved inactive in models of drug abuse [15].

In past years, FAAH inhibitor-induced endo-cannabinoid levels were shown to block cancer cell proliferation, including colorectal cancer cells [2] or to inhibit cancer growth *in vitro* and *in vivo*, e.g. of rat thyroid-transformed cells (KiMol) [16]. By contrast, the role of FAAH in tumor cell spreading is marginally (invasion) or not at all (metastasis) defined. The limited data published in this field were obtained using prostate carcinoma cells whose invasion was inhibited by 2-AG, a specific FAAH inhibitor or by siRNA targeting FAAH [17, 18]. The present study therefore investigated the impact of two FAAH inhibitors (URB597, AA-5HT) and four FAAH substrates (AEA, 2-AG, OEA, PEA) on lung cancer cell metastasis and invasion. Here we provide evidence for a pronounced antimetastatic action of both FAAH inhibitors that was mimicked by the FAAH substrates. Moreover, upregulation of tissue inhibitor of matrix metalloproteinases-1 (TIMP-1) was identified as molecular mechanism underlying the anti-invasive mechanism of both FAAH inhibitors.

## RESULTS

### Impact of FAAH inhibitors on levels of endocannabinoids and endocannabinoid-like substances

To prove the efficacy of the two FAAH inhibitors, AA-5HT and URB597, on the production of endocannabinoids and endocannabinoid-like substances by lung tumor cells, an LC-MS method was established allowing the simultaneous quantification of the FAAH substrates AEA, 2-AG, OEA and PEA [19]. Analyses of cell lysates from FAAH inhibitor-treated cells confirmed significantly increased intracellular levels of diverse FAAH substrates in the presence of either 10 μM AA-5HT or 10 μM URB597 as compared to vehicle (Table 1).

**Table 1:** Impact of the FAAH inhibitors AA-5HT and URB597 on levels of FAAH substrates in lung cancer cells

	A549				H460			
	Concentration FAAH substrate (pmol/mg protein)				Concentration FAAH substrate (pmol/mg protein)			
	AEA	2-AG	OEA	PEA	AEA	2-AG	OEA	PEA
Vehicle	0.80 ± 0.12	8.47 ± 1.43	3.88 ± 0.39	0.58 ± 0.13	0.89 ± 0.13	48.51 ± 5.80	6.84 ± 0.27	0.95 ± 0.19
AA-5HT (10 μM)	1.57 ± 0.21**	16.18 ± 1.77*	6.04 ± 0.75	0.99 ± 0.14	2.32 ± 0.41**	46.15 ± 5.34	13.17 ± 1.16***	2.08 ± 2.02**
URB597 (10 μM)	1.36 ± 0.11*	19.93 ± 3.24**	11.10 ± 2.07**	1.52 ± 0.33*	1.45 ± 0.22	89.18 ± 7.34***	12.76 ± 0.86***	1.56 ± 0.24

A549 and H460 cells were incubated with vehicle or with the respective FAAH inhibitor (each at 10 μM) for 6 h. Concentrations of FAAH substrates were quantified from cell lysates by LC-MS. All concentrations were normalized to cellular protein amounts. Values are means ± SEM of n = 9 experiments. *P < 0.05, **P < 0.01, ***P < 0.001 vs. vehicle; one-way ANOVA plus post hoc Dunnett test.

### Impact of FAAH inhibitors and FAAH substrates on tumor cell metastasis in nude mice

**Figure 1:**
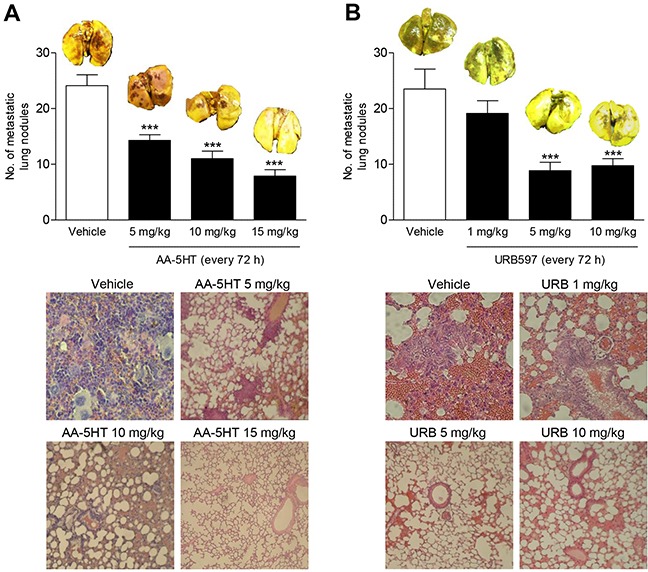
Impact of the FAAH inhibitors AA-5HT and URB597 on lung metastasis in nude mice A549 cells were injected intravenously in athymic nude mice. Mice were given intraperitoneal injections of AA-5HT **A.** and URB597 **B.** every 72 h for 28 days starting 24 h after injection of the cells. Complete lungs of animals sacrificed one day thereafter were evaluated for metastatic nodules after fixation in Bouin's fluid. Images above the histogram indicate Bouin's fluid-stained lungs; images below the histogram indicate hematoxylin/eosin stainings of paraffin sections from lungs. Values are means ± SEM of n = 7 - 9 (A) or n = 7 - 8 (B) animals per group. ***P < 0.001 vs. vehicle; one-way ANOVA plus post hoc Dunnett test.

To assess the impact of FAAH inhibitors on experimental metastasis, athymic nude mice were given intravenous injections of A549 lung cancer cells followed by a 4-week administration of AA-5HT and URB597, respectively. According to Figure 1A, AA-5HT caused a dose-dependent antimetastatic action. Thus, the numbers of metastatic nodules were significantly reduced in lungs of animals that were treated with AA-5HT at doses ≥ 5 mg/kg every 72 h. In case of URB597 a maximal reduction of metastasis was observed at a dose of 5 mg/kg, whereas no further reduction was observed after administration of 10 mg/kg (Figure 1B).

**Figure 2:**
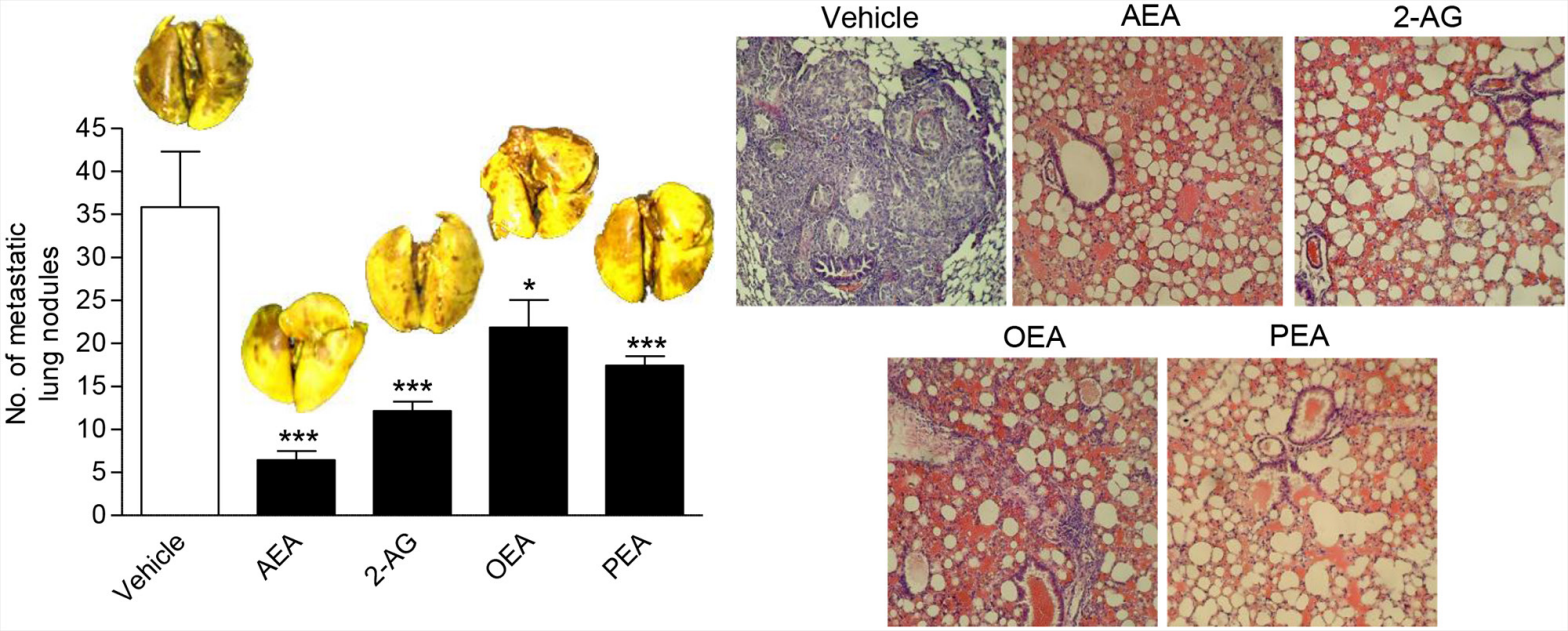
Impact of FAAH substrates on lung metastasis in nude mice A549 cells were injected intravenously in athymic nude mice. Mice were given intraperitoneal injections of AEA, 2-AG, OEA and PEA every 72 h for 28 days starting 24 h after injection of the cells. For all test substances, the initial dose (first administration) was 10 mg/kg followed by subsequent treatments with 5 mg/kg. Complete lungs of animals sacrificed one day thereafter were evaluated for metastatic nodules after fixation in Bouin's fluid. Images above the histogram indicate Bouin's fluid-stained lungs; images besides the histogram indicate hematoxylin/eosin stainings of paraffin sections from lungs. Values are means ± SEM of n = 6 - 7 animals per group. *P < 0.05, ***P < 0.001 vs. vehicle; one-way ANOVA plus post hoc Dunnett test.

In addition, an animal experiment using the endocannabinoids/endocannabinoid-like substances upregulated upon FAAH inhibition revealed an inhibition of metastasis by intraperitoneally injected AEA, 2-AG, OEA and PEA with AEA exhibiting the most pronounced antimetastatic effect (Figure 2).

### Impact of FAAH inhibitors on tumor growth in xenografted nude mice

**Figure 3:**
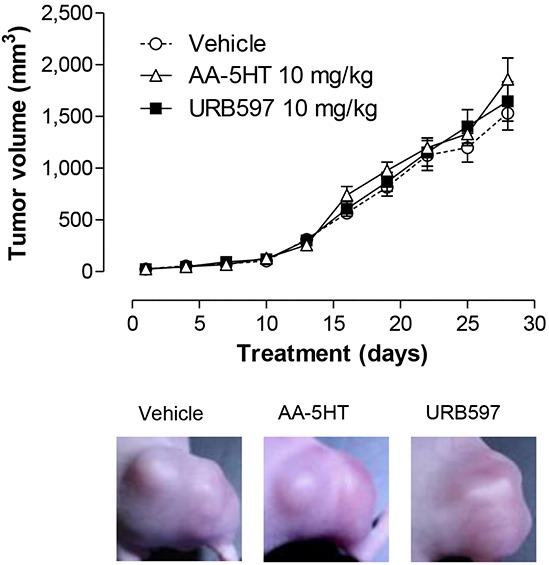
Impact of FAAH inhibitors on tumor growth in xenografted nude mice Tumors were generated by flank inoculation of A549 cells in nude mice. Animals were treated with either vehicle, AA-5HT (10 mg/kg i.p.) or URB597 (10 mg/kg i.p.) every 72 h for 28 days. Tumor sizes were measured with an external caliper and calculated as described in Materials and Methods. Images were taken from representative tumors on day 28. Tumor volumes are means ± SEM of n = 20 (vehicle) or n = 21 (AA-5HT, URB597) animals per group. ANOVA plus post hoc Dunnett test did not reveal significant differences between the vehicle- and AA-5HT- or URB597-treated groups.

To examine whether the antimetastatic effects of FAAH inhibitors on lung tumor cell metastasis were accompanied by tumor-regressive effects as shown for cannabidiol recently [20–22], the impact of AA-5HT and URB597 on the growth of tumors in A549-xenografted nude mice was investigated next. However, according to Figure 3, neither AA-5HT nor URB597 were found to reduce the tumor volume as compared to vehicle-treated animals.

### Impact of FAAH inhibitors on tumor cell invasion and TIMP-1 expression

**Figure 4:**
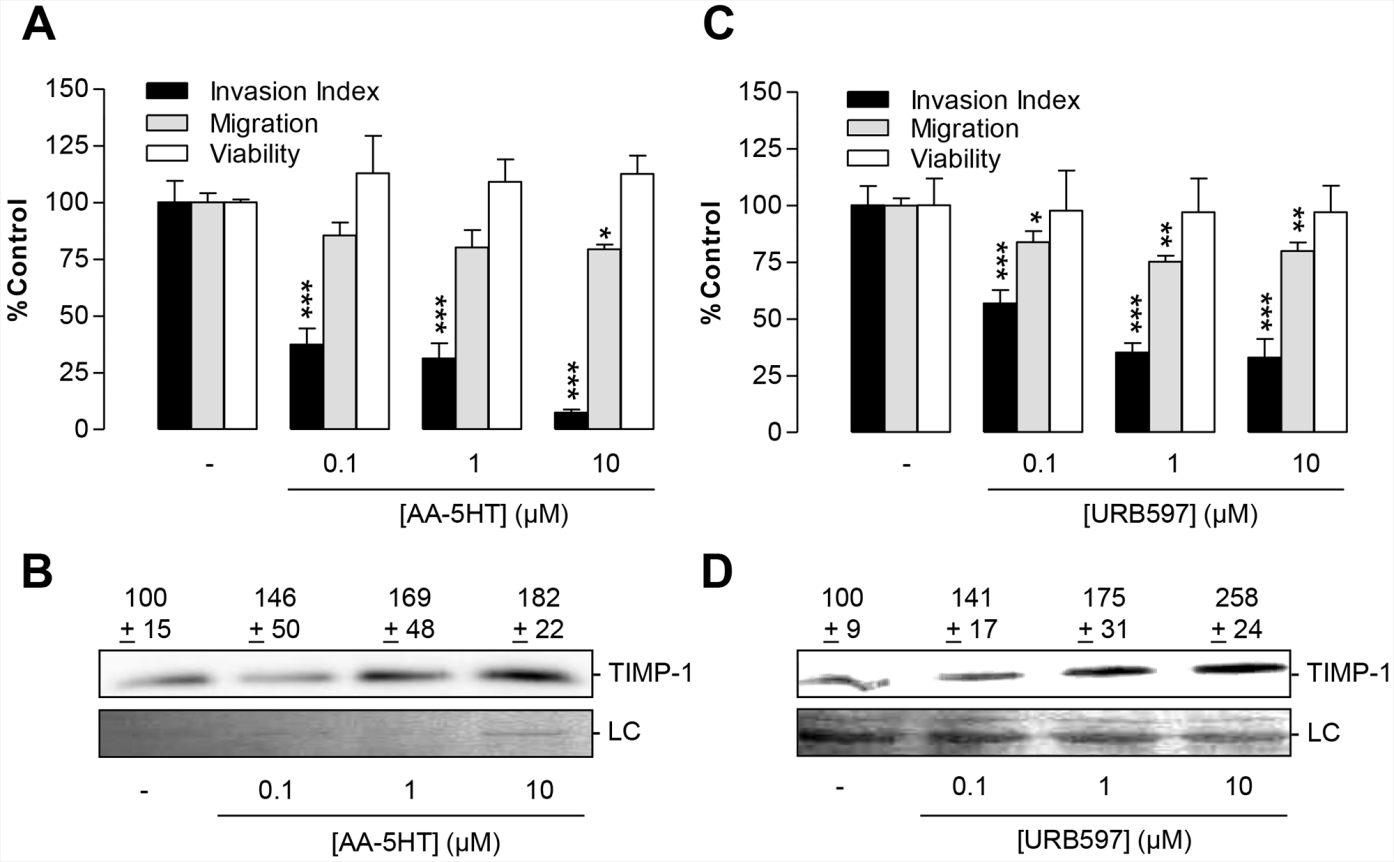
Impact of the FAAH inhibitors AA-5HT and URB597 on tumor cell invasion and TIMP-1 expression of A549 cells **A, C.** Concentration-dependency of FAAH inhibitors' anti-invasive action (black bars) and its impact on cell viability (open bars) and migration through uncoated Boyden chambers (grey bars). **B, D.** Concentration-dependent effect of FAAH inhibitors on TIMP-1 protein levels. A549 cells were incubated with vehicle, AA-5HT or URB597 for 72 h before measurement of invasion or migration using Boyden chamber assays (A, C), WST-1 test (A, C) or Western blot analyses of TIMP-1 from cell culture media (B, D). Protein staining of cell culture media is shown as loading control (LC). All values are expressed as percent control in comparison with vehicle-treated cells (100%) in the absence of test substance. Data are means ± SEM of n = 5 (A, invasion) or n = 4 (A,C migration and viability; B, blots), n = 11-12 (C, invasion) or n = 7 (D, blots) experiments. *P < 0.05, **P < 0.01, ***P < 0.001 vs. vehicle; one-way ANOVA plus post hoc Dunnett test.

To address a potential mechanism underlying the antimetastatic action of AA-5HT and URB597, the *in vitro* effect of either FAAH inhibitor on lung cancer cell invasion and levels of TIMP-1, an established anti-invasive mediator [23–25], was investigated next. In fact, both compounds were shown to confer a concentration-dependent anti-invasive (Figure 4A, 4C, black bars) and TIMP-1-inducing action (Figure 4B, 4D). In agreement with the protein data, increased TIMP-1 mRNA levels were assessed following a 48-h incubation with either FAAH inhibitor: AA-5HT (10 μM), 121% ± 11% vs. vehicle (100% ± 5%), means ± SEM of n = 20-21 experiments, *P* = 0.0886 vs. vehicle; URB597 (10 μM), 141% ± 11% vs. vehicle (100% ± 6%), means ± SEM of n = 21 experiments, **P < 0.01 vs. vehicle, Student's t test. However, compared to the densitometric analyses of the protein measurement by Western blots, the induction of TIMP-1 mRNA appeared rather weak.

Decreased invasion by both FAAH inhibitors was associated with a much smaller, but significant (AA-5HT at 10 μM, URB597 at 0.1 to 10 μM) decrease of migration through membranes that were not coated with Matrigel (Figure 4A, 4C, grey bars). On the other hand, cellular viability assessed under comparable conditions (5 × 10^5^ cells per 500 μl per well of a 48-well plate) was virtually unaltered in the presence of either FAAH concentration tested (Figure 4A, 4C, open bars).

### Effect of TIMP-1 knockdown on the anti-invasive action of FAAH inhibitors

**Figure 5:**
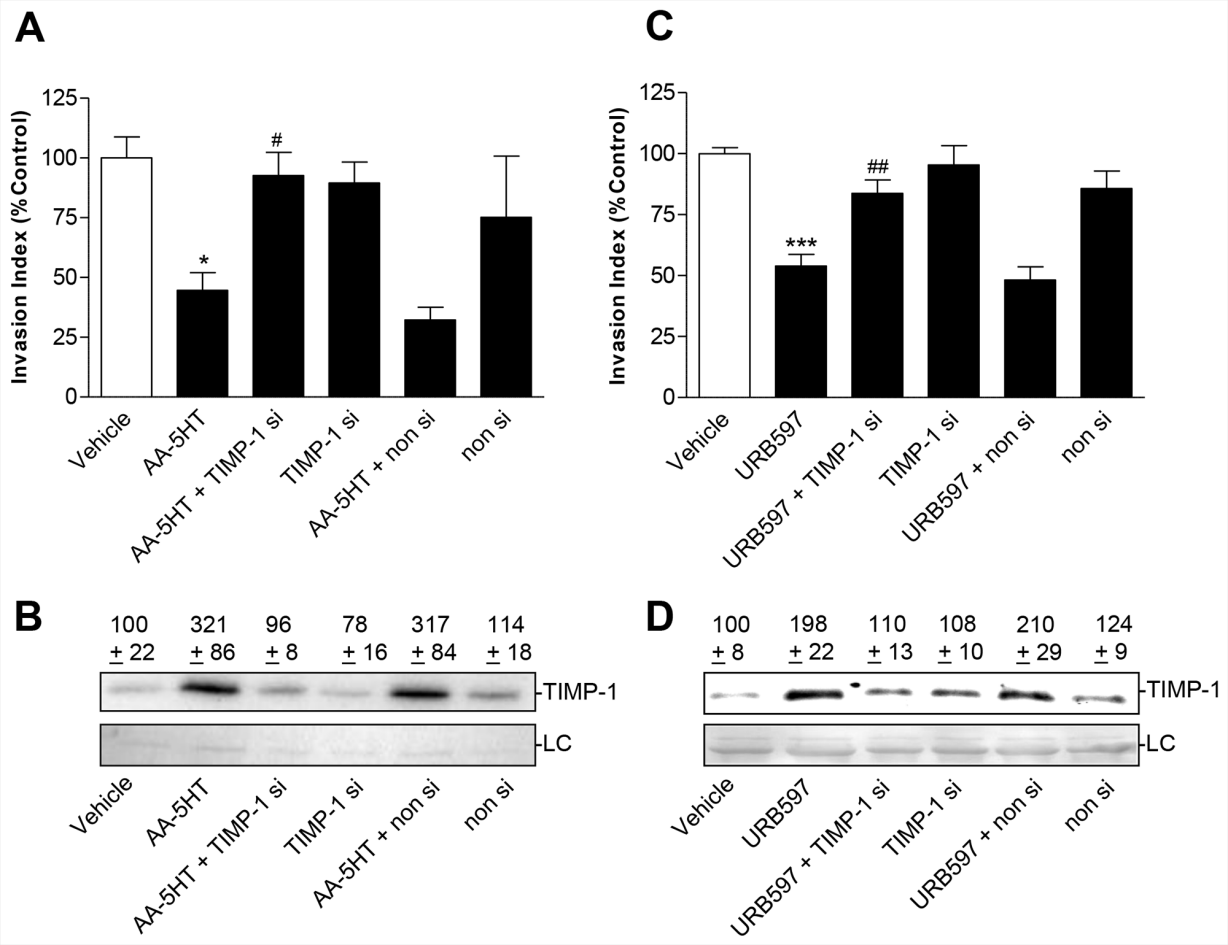
Effect of TIMP-1 knockdown on the anti-invasive and TIMP-1-upregulating action of FAAH inhibitors in A549 cells Effect of TIMP-1 siRNA on FAAH inhibitors' action on cell invasion **A, C.** and TIMP-1 protein levels from cell culture media **B, D.** A549 cells were transfected with TIMP-1 siRNA at a final concentration of 0.25 μg/ml siRNA (si) or with non-silencing siRNA (non si) for 24 h. Subsequently, cells were placed into invasion chambers in terms of invasion assays. Following the first transfection, cells were retransfected with the same amounts of TIMP-1 siRNA, non-silencing siRNA or suspension buffer to provide constant knockdown conditions, and incubated with vehicle, AA-5HT (10 μM) or URB597 (10 μM) for another 72 h. Protein staining of cell culture media is shown as loading control (LC). All values are expressed as percent control in comparison with vehicle-treated cells (100%) in the absence of test substance. Data are means ± SEM of n = 3-4 (A), n = 5 (B), n = 7-8 (C) or n = 6 (D) experiments. *P < 0.05, ***P < 0.001 vs. vehicle; ^#^*P* < 0.05, ^##^*P* < 0.01 vs. respective FAAH inhibitor; one-way ANOVA plus post hoc Bonferroni test.

To investigate a causal link between the FAAH inhibitor-mediated TIMP-1 induction and the accompanied decreased invasion, a specific siRNA targeting TIMP-1 was tested for its impact on FAAH-inhibitor-induced TIMP-1 expression and inhibition of invasiveness. According to previous investigations by our group, RNA interference with 0.25 μg/ml TIMP-1 siRNA elicited a reduction of cannabinoid-stimulated TIMP-1 expression without substantially affecting basal TIMP-1 expression [26, 27]. As shown in Figure 5A, 5C, knockdown of TIMP-1 expression led to an almost complete inhibition of the anti-invasive effect of either FAAH inhibitor, whereas cultures treated with a non-silencing siRNA control exhibited no significantly altered invasion patterns as compared to controls treated with transfection agent only. Analysis of TIMP-1 levels confirmed a complete (AA-5HT) or almost complete (URB597) inhibition of FAAH inhibitor-stimulated TIMP-1 formation in cells incubated with FAAH inhibitor and additionally transfected with TIMP-1 siRNA (Figure 5B, 5D). Again, the non-silencing control left TIMP-1 levels upregulated by either FAAH inhibitor virtually unaltered.

### Impact of cannabinoid receptor- and TRPV1 antagonists on the anti-invasive and TIMP-1-inducing action of FAAH inhibitors

Recently, the expression of the cannabinoid receptors CB_1_ and CB_2_ as well as of TRPV1 was assessed in various lung cancer cell lines (A549, H358, H460) as well as primary lung cancer cells by Western blot analyses of membrane fractions [21]. To investigate whether the anti-invasive action elicited upon FAAH inhibition was a result of activation of either of these receptors, the impact of antagonists to CB_1_ (AM-251), CB_2_ (AM-630) and TRPV1 (capsazepine) on invasiveness and TIMP-1 expression by A549 cells was investigated next. All antagonists were used at a concentration of 1 μM, which has been reported to be within the range of concentrations inhibiting CB_1_-, CB_2_- and TRPV1-dependent events [20, 26–31].

**Figure 6:**
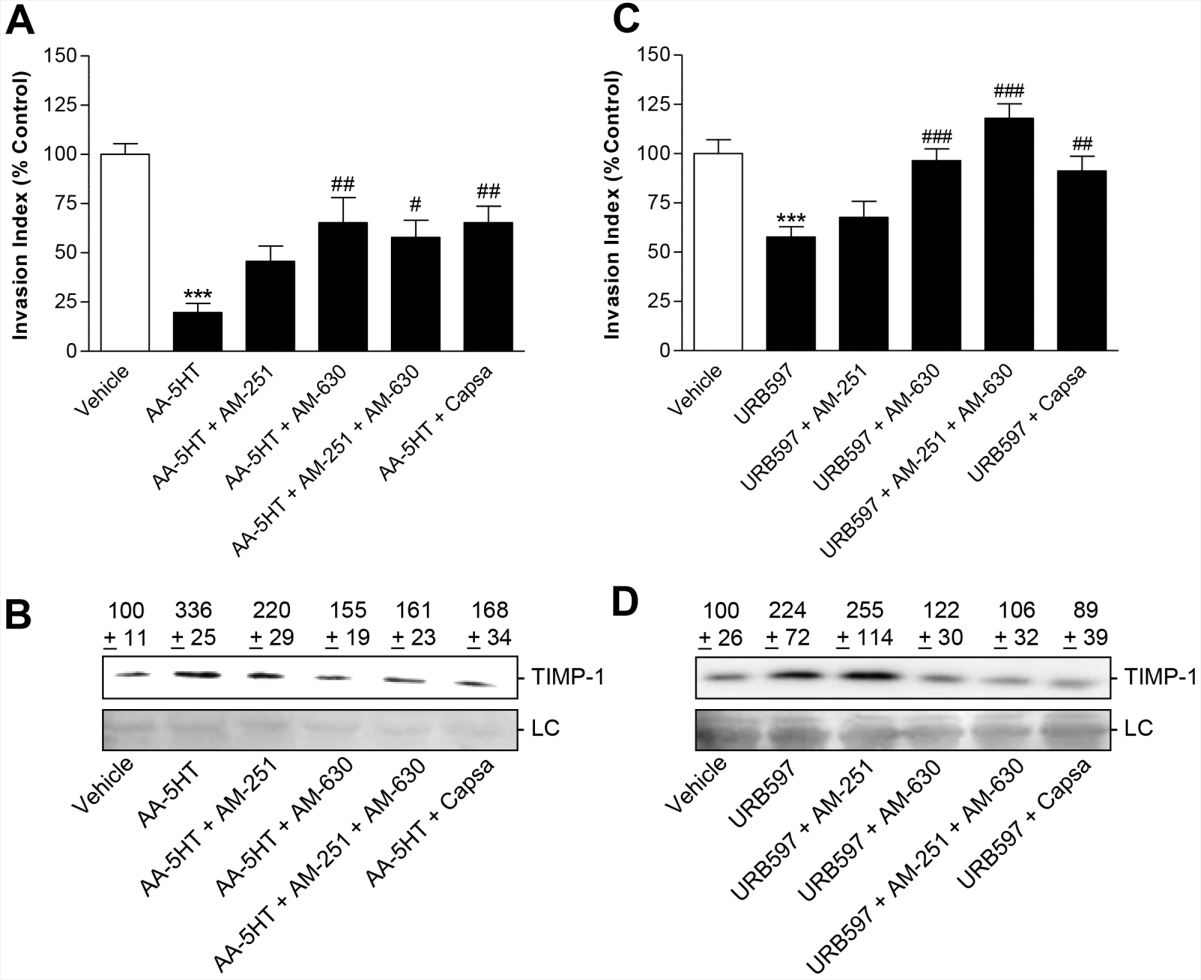
Impact of cannabinoid receptor- and TRPV1 antagonists on the anti-invasive and TIMP-1-inducing action of FAAH inhibitors in A549 cells A549 cells were preincubated with AM-251 (CB_1_ antagonist; 1 μM), AM-630 (CB_2_ antagonist; 1 μM) or capsazepine (capsa; TRPV1 antagonist; 1 μM) for 1 h. Following addition of vehicle, AA-5HT (10 μM) or URB597 (10 μM) incubation was continued for another 72 h before measurement of invasion using Matrigel Boyden chamber assays **A, C.** or Western blot analyses of TIMP-1 in cell culture media **B, D.** Protein staining of cell culture media is shown as loading control (LC). All values are expressed as percent control in comparison with vehicle-treated cells (100%) in the absence of test substance. Data are means ± SEM of n = 9-12 (A), n = 6 (B), n = 11-12 (C) or n = 3 (D) experiments. ***P < 0.001 vs. vehicle; ^#^*P* < 0.05, ^##^*P* < 0.01, ^###^*P* < 0.001 vs. respective FAAH inhibitor; one-way ANOVA plus post hoc Bonferroni test.

In these experiments, antagonists to the CB_2_ receptor and to TRPV1 suppressed the anti-invasive action of AA-5HT and URB597 in A549 cells in a statistically significant manner (Figure 6A, 6C). In line with this notion, the same receptor antagonists diminished increased TIMP-1 protein levels in cells incubated with the respective FAAH inhibitor (Figure 6B, 6D). In case of URB597, the inhibitory effect of AM-630 on both invasion and TIMP-1 was even increased, when cells were incubated with a combination of CB_2_ (AM-630) and CB_1_ (AM-251) antagonists (Figure 6C, 6D).

### Impact of FAAH knockdown on tumor cell invasion – role of cannabinoid-activated receptors

**Figure 7:**
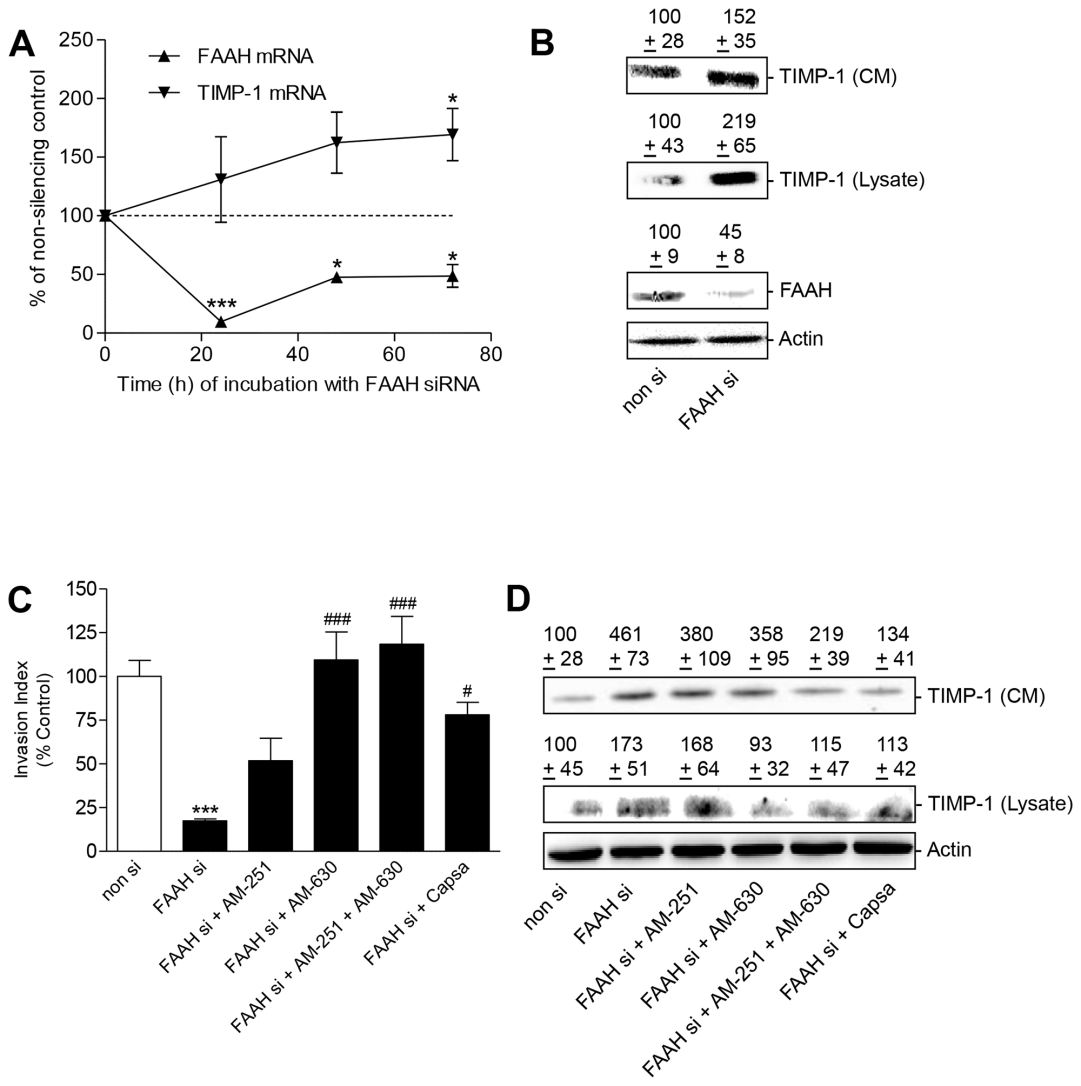
Impact of FAAH knockdown on tumor cell invasion and TIMP-1 expression - role of cannabinoid-activated receptors **A.** Time-courses of FAAH- and TIMP-1 mRNA expression following knockdown of FAAH. A549 cells were incubated with 2.5 μg/ml FAAH siRNA or a non-silencing siRNA (non si, dashed line) for the indicated times. **B.** TIMP-1 and FAAH protein expression was measured by Western blots of cell lysates and cell culture media (CM, upper blots). For analysis of protein levels A549 cells were incubated with 2.5 μg/ml siRNA or a non-silencing siRNA for 72 h. Values above the Western blots indicate means ± SEM obtained from densitometric analyses. **C, D.** Impact of antagonists of cannabinoid-activated receptors on the anti-invasive and TIMP-1-inducing action of FAAH siRNA. A549 cells were incubated with 2.5 μg/ml siRNA or a non-silencing siRNA for 72 h in the presence or absence of receptor antagonists (all at 1 μM) to CB_1_ (AM-251), CB_2_ (AM-630) and TRPV1 (capsazepine). Values are means ± SEM of n = 4 (A, FAAH mRNA, 24 and 48 h; B, TIMP-1 blots), n = 3 (A, TIMP-1 mRNA, 48 h; C,D), n = 8 (A, FAAH mRNA, 72 h; A, TIMP-1 mRNA, 24 h), n = 9 (B, FAAH blots) or n = 10 (A, TIMP-1 mRNA, 72 h) experiments. *P < 0.05, ***P < 0.001 vs. non-silencing siRNA-transfected cells, ^#^*P* < 0.05, ^###^*P* < 0.001 vs. FAAH siRNA-transfected cells; ANOVA plus post hoc Bonferroni test (C) or Student's t test (A).

To exclude unspecific effects of the FAAH inhibitors, A549 cells were tested for the effect of FAAH knockdown on cell invasion and TIMP-1 expression using siRNA. As shown in Figure 7, knock-down of FAAH by specific siRNA was associated with a profound inhibition of FAAH expression and increased TIMP-1 expression on both mRNA (Figure 7A) and protein level (Figure 7B). As compared to the substantial downregulation of FAAH mRNA in response to FAAH siRNA, the subsequent induction of TIMP-1 mRNA occurred delayed reaching statistical significance even following a 72-h incubation with FAAH siRNA (Figure 7A). In line with the results obtained from experiments using FAAH inhibitors reported before, the anti-invasive effect of FAAH siRNA became significantly reversed following single blockade of CB_2_ or TRPV1 as well combined blockade of CB_1_ and CB_2_ (Figure 7C). By contrast, in the presence of a non-silencing siRNA control, none of the receptor antagonists did alter basal invasiveness significantly. The respective invasion rates were as follows: non-silencing siRNA, 100% ± 11%; non-silencing siRNA + AM-251 (1 μM), 96% ± 8%; non-silencing siRNA + AM-630 (1 μM), 99% ± 11%; non-silencing siRNA + AM-251 (1 μM) + AM-630 (1 μM), 96% ± 14%; non-silencing siRNA + capsazepine (1 μM), 78% ± 8%, means ± SEM of n = 4 experiments. A significant difference versus the non-silencing siRNA control was not determinable for any treatment group using ANOVA plus post hoc Bonferroni test.

In line with the invasion data, receptor antagonists to CB_2_ or TRPV1 diminished FAAH siRNA-induced TIMP-1 protein levels in cell culture media (Figure 7D, upper blot). Inhibition of TIMP-1 expression by FAAH siRNA was likewise found when analyzing cellular lysates with CB_2_ blockade conferring a complete and combined CB_1_/CB_2_ blockade or TRPV1 antagonism eliciting an almost complete reversal of TIMP-1 increases (Figure 7D, lower blot).

### Impact of the FAAH substrates AEA, 2-AG, OEA and PEA on tumor cell invasion and TIMP-1 levels

**Figure 8:**
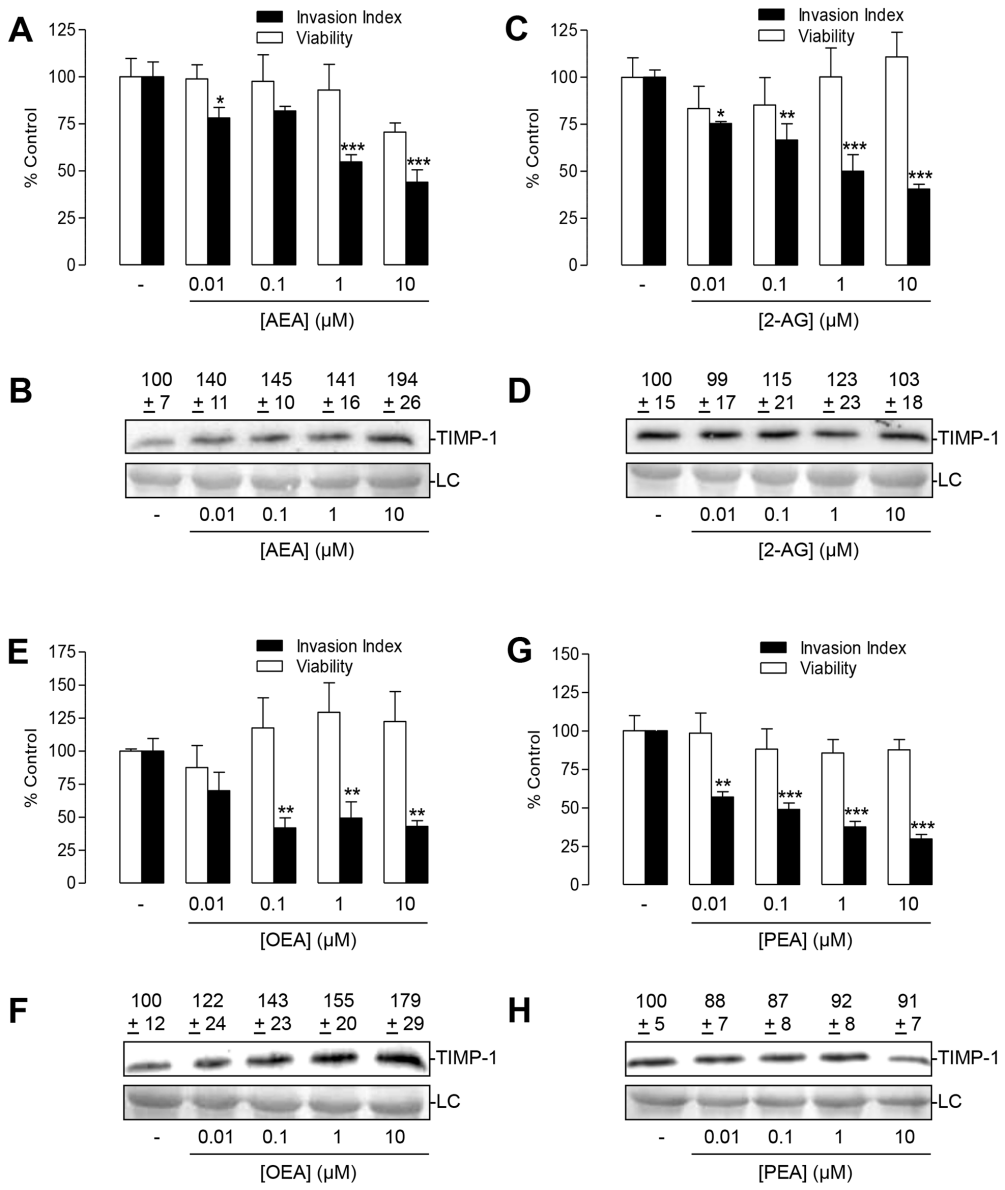
Impact of the FAAH substrates AEA, 2-AG, OEA and PEA on tumor cell invasion and TIMP-1 secretion of A549 cells **A, C, E, G.** Concentration-dependency of FAAH substrates' anti-invasive action (black bars; Boyden chamber assays) and its impact on cell viability (open bars, WST-1 test) following a 72-h incubation period. **B, D, F, H.** Concentration-dependent effect of FAAH substrates on TIMP-1 protein levels following a 72-h incubation period. Protein staining of cell culture media is shown as loading control (LC). Numbers above the blots (B, D, F, H) represent results of densitometric analyses. All values are expressed as percent control in comparison with vehicle-treated cells (100%) in the absence of test substance. Data are means ± SEM of n = 4 (A,C,G, invasion, viability; E, viability; B;F), n = 7-8 (E, invasion) or n = 8 (D, H) experiments. *P < 0.05, **P < 0.01, ***P < 0.001 vs. vehicle; one-way ANOVA plus post hoc Dunnett test.

Based on the detected elevation of endocannabinoids and endocannabinoid-like substances in FAAH inhibitor-treated lung cancer cells, a potential anti-invasive and TIMP-1-upregulating effect of exogenously added AEA, 2-AG, OEA and PEA was investigated next. Analysis of AEA, 2-AG, OEA and PEA, tested in a range from 0.01 to 10 μM in A549 cells, revealed a concentration-dependent anti-invasive effect of either FAAH substrate (Figure 8A, 8C, 8E, 8G, black bars). Cellular viability measured under comparable conditions (5 × 10^5^ cells per 500 μl per well of a 48-well plate) did not reveal statistically significant changes in the presence of either FAAH substrate (Figure 8A, 8C, 8E, 8G, open bars).

However, a concentration-dependent upregulation of TIMP-1 protein levels was only observed in culture supernatants of cells incubated for 72 h with AEA (Figure 8B) and OEA (Figure 8F). On the other hand, PEA (Figure 8H) did not upregulate TIMP-1 levels and 2-AG (Figure 8D) exhibited only a weak TIMP-1 increase.

**Figure 9:**
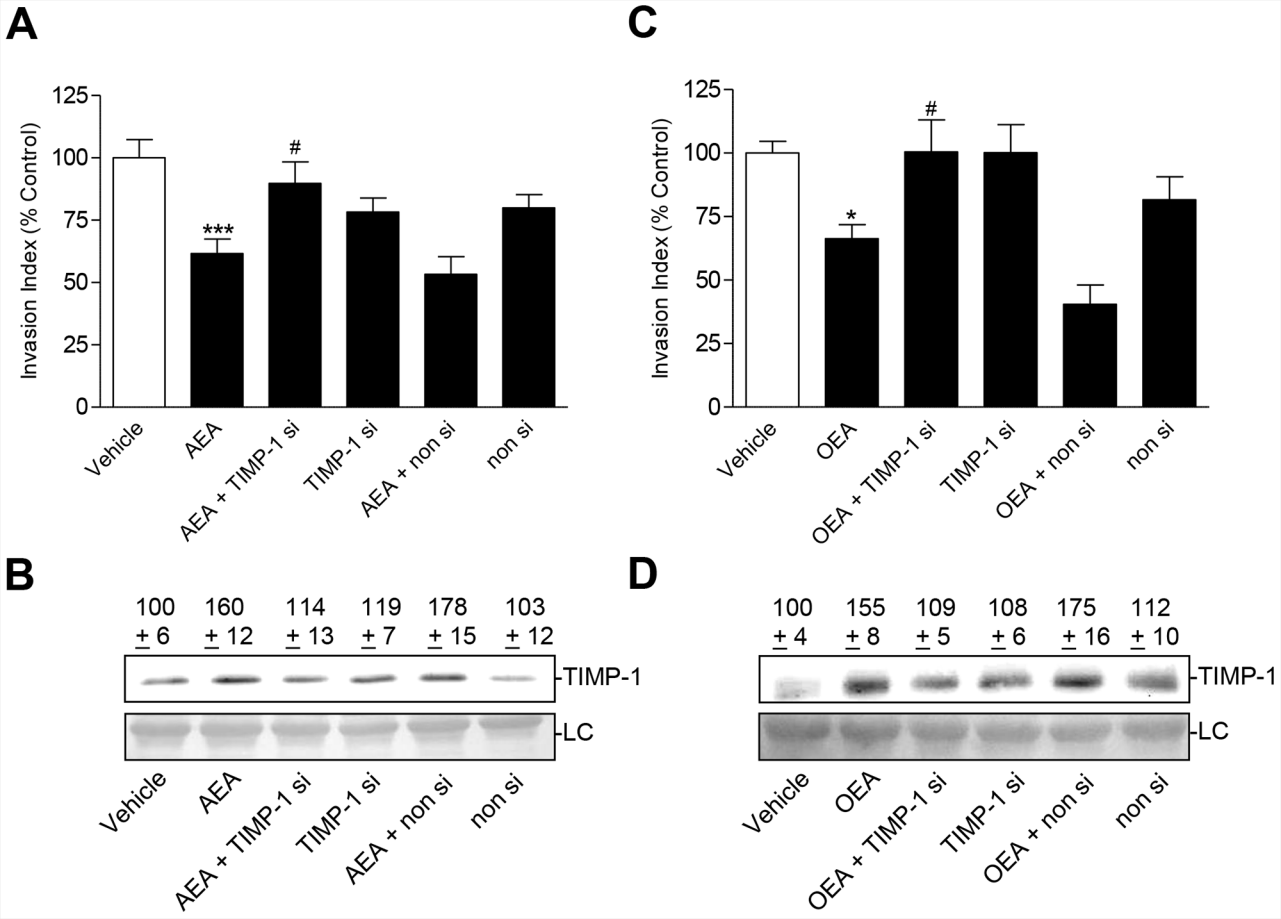
Effect of TIMP-1 knockdown on the anti-invasive and TIMP-1-upregulating action of AEA and OEA in A549 cells Effect of TIMP-1 siRNA on the anti-invasive **A, C.** and TIMP-1-upregulating action **B, D.** of AEA and OEA, respectively. A549 cells were transfected with TIMP-1 siRNA at a final concentration of 0.25 μg/ml siRNA (si) or with non-silencing siRNA (non si) for 24 h. Subsequently, cells were placed into invasion chambers in terms of invasion assays. Following the first transfection, cells were retransfected with the indicated type of siRNA or suspension buffer to provide constant knockdown conditions, and incubated with vehicle, AEA (10 μM) or OEA (10 μM) for another 72 h. Protein staining of cell culture media is shown as loading control (LC). Numbers above the blots (B,D) represent densitometric analyses of the respective Western blots. All values are expressed as percent control in comparison with vehicle-treated cells (100%) in the absence of test substance. Data are means ± SEM of n = 11-12 (A), n = 6 (B), n = 15-16 (C) or n = 10 (D) experiments. *P < 0.05, ***P < 0.001 vs. vehicle; ^#^*P* < 0.05 vs. AEA or OEA; one-way ANOVA plus post hoc Bonferroni test.

As shown for the FAAH inhibitors before, knockdown of upregulated TIMP-1 expression (Figure 9B, 9D) led to a significant inhibition of the anti-invasive effect of AEA (Figure 9A) and OEA (Figure 9C). Again, invasion of cultures treated with a non-silencing siRNA control did not significantly differ from invasion of cells treated with transfection agent only (Figure 9A, 9C).

### Impact of FAAH inhibitors on invasion and TIMP-1 induction in other lung cancer cells

**Figure 10:**
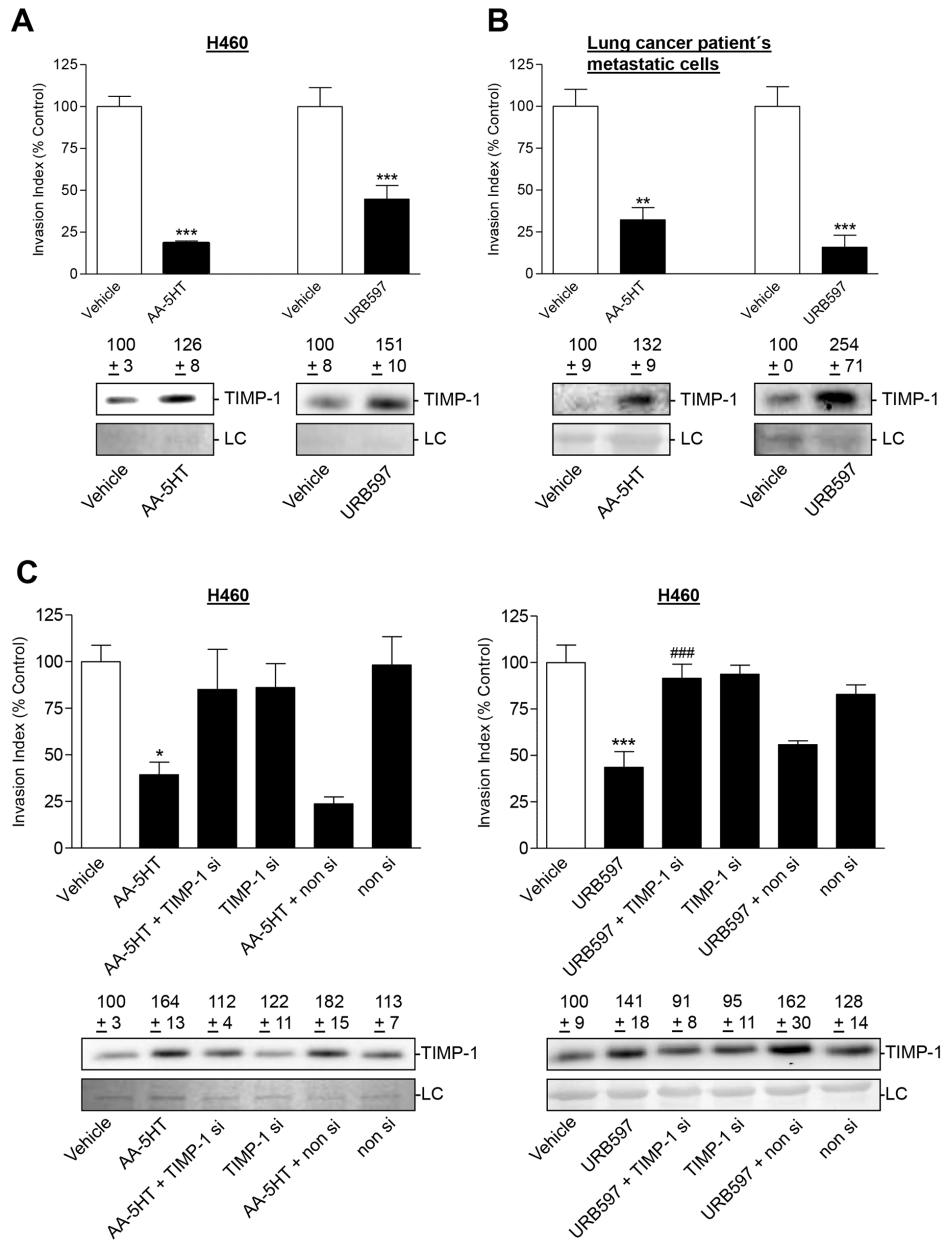
Effect of TIMP-1 knockdown on the anti-invasive and TIMP-1-upregulating action of FAAH inhibitors in other lung cancer cells **A, B.** Effect of AA-5HT (10 μM) and URB597 (10 μM) on invasion and TIMP-1 expression in the lung cancer cell line H460 (A) and lung cancer patient's metastatic cells (B) following a 72-h incubation period, respectively. **C.** Effect of TIMP-1 siRNA on FAAH inhibitors' action on cell invasion and TIMP-1 protein levels in H460 cells. H460 cells were transfected with TIMP-1 siRNA at a final concentration of 0.25 μg/ml siRNA (si) or with non-silencing siRNA (non si) for 24 h. Subsequently, cells were placed into invasion chambers in terms of invasion assays. Following the first transfection, cells were retransfected with the indicated type of siRNA or suspension buffer to provide constant knockdown conditions, and incubated with vehicle, AA-5HT (10 μM) or URB597 (10 μM) for another 72 h. Protein staining of cell culture media is shown as loading control (LC). Numbers above the blots represent densitometric analyses of the respective Western blots. All values are expressed as percent control in comparison with vehicle-treated cells (100%) in the absence of test substance. Data are means ± SEM of n = 3 (A, left blot; B, both blots; C left blot), n = 3-4 (C, right panel, invasion), n = 4 (B, invasion), n = 6 (A, right blot; C right blot), n = 7-8 (C, left panel, invasion), n = 8 (A, left panel, invasion) or n = 12 (A, right panel, invasion) experiments. *P < 0.05, **P < 0.01, ***P < 0.001 vs. vehicle; ^###^*P* < 0.001 vs. URB597; ANOVA plus post hoc Bonferroni test (C) or Student's t test (A, B).

To exclude that the demonstrated effects are restricted to A549 cells, key experiments were also performed in other human lung cancer cells. Like in A549, incubation of H460 or lung cancer patient's metastatic cells with either FAAH inhibitor resulted in a profound suppression of invasion through Matrigel-coated transwell plates (Figure 10A, 10B, upper panels). Decreased invasiveness was accompanied by increased TIMP-1 secretion (Figure 10A, 10B, Western blots, lower panels). RNA interference experiments revealed transfection of H460 with TIMP-1 siRNA to cause inhibition of both anti-invasive (Figure 10C, upper panels) as well as TIMP-1-upregulating effects of FAAH inhibitors (Figure 10C, Western blots, lower panels), whereas a non-silencing control was virtually inactive in this respect.

## DISCUSSION

Taken into account that death from almost all fatal cancer diseases results from metastasis, there is currently a clinical need for new pharmacotherapeutical options to treat malignant cancers. On the basis of data obtained from animal experiments published during the last decade, cannabinoids have gained interest as a considerable option for the treatment of metastatic cancers (for review see [1]).

The present study provides first-time proof for an antimetastatic action of FAAH inhibitors with a substantial 67% and 62% inhibition of metastasis following repeated administration of 15 mg/kg AA-5HT and 5 mg/kg URB597, respectively. Although a bench-to-bedside conversion remains to be elucidated in clinical studies, a literature search revealed the observed antimetastatic effect of FAAH inhibitors to be comparable to that of commonly used chemotherapeutic drugs. Accordingly, taxol elicited a 35% reduction of breast cancer metastasis in lungs of mice [32], 5-fluorouracil caused an approximate bisection of the total number of peritoneal nodules in a mouse metastasis model using esophageal squamous carcinoma cells [33], and cisplatin in 2 of 5 animals failed to inhibit metastasis in an orthotopic metastatic nude mouse model of oral tongue squamous cell carcinoma [34]. In addition, the effect of FAAH inhibition on tumor metastasis of comparable lung cancer models was even superior to thalidomide [35]. With respect to other cannabinoids, previous investigations of our group using the same experimental model as in the present study revealed an 84% [27] or 52% [21] inhibition of lung cancer metastatic nodules in athymic nude mice treated with 5 mg/kg cannabidiol, a non-psychoactive phytocannabinoid.

On the other hand, a tumor-regressive action in A549-xenografted nude mice as shown for cannabidiol recently [20–22] was not observed with AA-5HT and URB597. In line with this finding, URB597, given at 1 mg/kg every third day for three weeks [36] or at 10 mg/kg daily for six days [37], failed to suppress tumor growth in mice xenografted with H460 lung cancer [36] and melanoma cells [37], respectively. In these studies inhibition of tumor growth was even observed when URB597 was combined with a synthetic analogue of AEA [36] or with PEA [37]. On the other hand, Bifulco et al. [16] using athymic mice xenografted with rat thyroid transformed (KiMol) cells reported a tumor-regressive action of AA-5HT, given at repeated doses of 5 mg/kg, implying a tumor cell-specific action of this FAAH inhibitor.

To address a potential mechanism underlying the antimetastatic action of FAAH inhibitors, the anti-invasive properties of AA-5HT and URB597 were focussed on in further experiments. As a result of these approaches, both FAAH inhibitors were shown to confer anti-invasive effects via upregulation of the matrix metalloproteinase (MMP) inhibitor TIMP-1. There are several lines of evidence supporting this notion. First, either FAAH inhibitor caused a concentration-dependent anti-invasive action that was accompanied by a likewise concentration-dependent upregulation of TIMP-1. Noteworthy, none of the FAAH inhibitors tested elicited a cytotoxic response on A549 under similar conditions, thus excluding an unspecific toxicity-related phenomenon. Second, post-transcriptional knock-down of FAAH inhibitor-induced TIMP-1 expression by specific siRNA was found to abrogate decreased invasiveness, thereby substantiating TIMP-1 to mediate the anti-invasive action of FAAH inhibitors. Third, FAAH was proven to be functionally involved in invasiveness and TIMP-1 expression by findings demonstrating an anti-invasive and TIMP-1-upregulating impact of FAAH knockdown with the respective siRNA. Fourth, anti-invasive and TIMP-1-upregulating properties of AA-5HT and URB597 were confirmed in another lung tumor cell line, H460, as well as in metastatic cells obtained from resection of brain metastasis of a patient with non-small cell lung cancer (NSCLC), indicating the observed effects to be not restricted to one cell line.

In addition to these mechanistic insights, there are several lines of evidence indicating biologically active FAAH substrates to confer the effects of FAAH inhibitors shown here. Thus, LC-MS analyses revealed increased levels of diverse FAAH substrates in A549 and H460 cells incubated with either FAAH inhibitor. Following administration to nude mice or addition to A549 cells, the FAAH substrates AEA, 2-AG, OEA and PEA mimicked the antimetastatic and anti-invasive action of FAAH inhibitors. Finally, a complete or partial reversal of the anti-invasive and TIMP-1-upregulating action of either FAAH inhibitor or FAAH siRNA was achieved when cannabinoid receptors (CB_1_, CB_2_) or TRPV1 were blocked with specific antagonists. Concerning the contribution of particular FAAH substrates, it is tempting to speculate that AEA and OEA, both mimicking the concentration-dependent increase of TIMP-1 by the FAAH inhibitors, may play a pivotal role in this response. In line with this notion, a knockdown of upregulated TIMP-1 expression by either FAAH substrate led to inhibition of the anti-invasive effect of AEA and OEA. In contrast to AEA and 2-AG, OEA as well as PEA do not bind to the cannabinoid receptors CB_1_ and CB_2_, but share an activation of TRPV1 with the two endocannabinoids. However, given that the FAAH substrates 2-AG and PEA likewise suppressed cancer cell invasion without (PEA) or only weakly (2-AG) increasing TIMP-1 following a 72-h incubation, additional anti-invasive mechanisms of FAAH inhibitors appear feasible.

As a matter of fact, the impact of endocannabinoid signaling on tumor cell invasion is poorly defined. A few data published in this field indicate an anti-invasive action of 2-AG in prostate carcinoma cells [17, 18] which contain comparatively high 2-AG and low AEA levels [17]. In addition, anti-invasive properties on prostate carcinoma cells have been described for CAY10401, a specific FAAH inhibitor, and for FAAH siRNA [18]. The present study therefore provides first-time proof for a mechanism underlying the anti-invasive action of an FAAH inhibitor. The observation that TIMP-1 plays a pivotal role in this response is corroborated by several investigations that found a correlation between cancer cell invasion and decreased TIMP-1 levels [23–25]. The inhibitory action of TIMPs on collagen-degrading MMPs determines the proteolytic activity of tumor tissues during cancer progression, thereby regulating tumor cell invasion, metastasis and angiogenesis [38, 39]. The hypothesis of TIMP-1 as crucial inhibitor of proteolytic properties conferring the anti-invasive action is substantiated by our findings showing FAAH inhibitors to only slightly inhibit the migration of A549 cells through uncoated Boyden chambers. Concerning the impact of cannabinoids on cancer cell spreading, previous findings of our group indicate the anti-invasive effects of several cannabinoids, including R(+)-methanandamide, Δ^9^-tetrahydrocannabinol as well as cannabidiol to be causally linked to TIMP-1 induction via a mechanism involving activation of cannabinoid receptors and TRPV1 [21, 26, 27].

While performing this study, another investigation was published showing no impact of URB597 (0.2 μM) and Met-F-AEA (10 μM), a stable AEA analogue, on the invasion of lung cancer cells that was induced by epidermal growth factor [36]. In view of this data which are differing from the outcome of the present study, it is conceivable that the anti-invasive action of URB597 and AEA depends on details of the experimental setting such as the activation status of the cancer cells.

Collectively, the present study provides the first evidence for an antimetastatic action of FAAH inhibitors as well as for a TIMP-1-dependent mechanism underlying its anti-invasive properties. In view of current clinical demands, a further research on the antimetastatic properties of these substances and its endogenous substrates may open an exciting field in search for novel treatment options for malignant cancers.

## MATERIALS AND METHODS

### Materials

AA-5HT, 2-AG, AM-251, AM-630, OEA, PEA and URB597 were obtained from Biomol GmbH (Hamburg, Germany). AEA was purchased from Enzo Life Sciences GmbH (Lörrach, Germany). Dimethyl sulfoxide (DMSO), ethylenediaminetetraacetic acid (EDTA), glycerol, glycine, hydrogen peroxide (H_2_O_2_), sodium chloride (NaCl), Tris hydrocloride (Tris-HCl) and Tris ultrapure were obtained from AppliChem (Darmstadt, Germany). 4-(2-hydroxyethyl)-1-piperazineethanesulfonic acid (HEPES) was bought from Ferak (Berlin, Germany). Aprotinine, bovine serum albumin, capsazepine, HCl, leupeptine, luminal, orthovanadate, para-coumaric acid, para-formaldehyde, phenylmethylsulfonyl fluoride (PMSF) and Triton^®^ X-100 were from Sigma (Taufkirchen, Germany). Dulbecco's modified eagle medium (DMEM) with 4.5 g/l glucose and with L-glutamine was provided by Lonza (Cologne, Germany). Fetal calf serum (FCS) and penicillin-streptomycin were purchased from Invitrogen (Darmstadt, Germany) and phosphate-buffered saline (PBS) was provided by PAN Biotech (Aidenbach, Germany). Milk powder was obtained from Bio-Rad Laboratories GmbH (Munich, Germany). Acrylamide (Rotiphorese^®^ Gel 30) was obtained from Carl Roth GmbH (Karlsruhe, Germany). Pure standards for LC-MS analysis of AEA, 2-AG, OEA, PEA and AEA-d8 were purchased from Cayman Chemical (Ann Arbor, MI, USA).

### Cell culture

A549 and H460 lung carcinoma cells were maintained in DMEM supplemented with 10% heat-inactivated FCS, 100 U/ml penicillin and 100 μg/ml streptomycin. Lung cancer patient's metastatic cells were obtained from resection of brain metastasis of a 67-year-old male Caucasian with NSCLC with the procedure of cell preparation described recently [21]. The patient had been informed about the establishment of cellular models from its tumor and had given informed consent in written form. For these experiments, cells were passaged 5–7 times without intermediate freezing steps in DMEM containing 20% FCS and 100 U/ml penicillin and 100 μg/ml streptomycin. The procedure was approved by the local Ethics Committee.

All incubations were performed in serum-free DMEM. PBS was used as vehicle for test substances with a final concentration of 0.1% (v/v) DMSO (for AA-5HT, URB597, AM-251, AM-630, capsazepine) or 0.1% (v/v) ethanol (for AEA, 2-AG, OEA, PEA). PBS containing the respective concentration of DMSO or ethanol was used as vehicle control.

### Matrigel invasion assay

The invasiveness of cells was quantified using a modified Boyden chamber technique with Matrigel-coated membranes according to the manufacturer's instructions (BD Biosciences, Oxford, UK) as described recently [26, 27, 40]. In this assay, cells must overcome a reconstituted basement membrane by proteolytic degradation of a Matrigel layer and active migration. In brief, the upper sides of the transwell inserts (8-μm pore size) were coated with 28.4 μg Matrigel per insert in a 24-well plate format. Cells were used at a final concentration of 5 × 10^5^ cells per well in a volume of 500 μl serum-free DMEM in each insert and treated with test substances or vehicles for the indicated times. DMEM containing 10% FCS was used as a chemoattractant in the companion plate. Following incubation in a humidified incubator at 37°C and 5% CO_2_ for the indicated times, the non-invading cells on the upper surface of the inserts were removed with a cotton swab, and viability of invaded cells on the lower surface was measured by the colorimetric WST-1 test (4-[3-(4-Iodophenyl)-2-(4-nitrophenyl)-2H-5-tetrazolio]-1.3-benzene disulfonate; Roche Diagnostics, Mannheim, Germany). For calculation of migration, the viability of cells on the lower side of uncoated invasion chambers was determined by the WST-1 test. Invasion was expressed as the invasion index, which is calculated as the absorbance at 450 nm with a reference filter at 690 nm of cells that invaded through Matrigel-coated Boyden chambers divided by absorbance of cells that migrated through uncoated control inserts with equal treatment ([invasion/migration] x 100%).

### Analysis of cytotoxicity

To exclude the possibility that the anti-invasive action of FAAH inhibitors and its substrates was an unspecific cytotoxicity-related phenomenon, cell viability was analyzed after exposure with either substance. To match the conditions of the invasion assays, cells were seeded into 48-well plates at 5 × 10^5^ cells per well in a volume of 500 μl DMEM per well. Thereafter, cells were immediately treated with test substance or vehicle for another 72 h. Viability was measured subsequently using the WST-1 test (Roche Diagnostics).

### Quantitative reverse-transcriptase polymerase chain reaction (RT-PCR) analysis

Cells were seeded into 24-well plates at a density of 1 × 10^5^ cells per well and were grown for 24 h in DMEM containing 10% FCS. Following incubation with the respective compound or its vehicle for the indicated times, cell culture media were removed and cells were lysed for subsequent RNA isolation using the RNeasy total RNA Kit (Qiagen, Hilden, Germany). β-Actin- (internal standard), FAAH- and TIMP-1 mRNA levels were determined by quantitative real-time RT-PCR as described [41]. Primers and probes for human β-actin, FAAH and TIMP-1 were TaqMan^®^ Gene Expression Assays (Applied Biosystems, Darmstadt, Germany).

### Western blot analysis

TIMP-1 was determined in cell culture media collected from upper Boyden chambers, except Figures 4B, 5B, and Figure 7. In Figure 4B and 5B, TIMP-1 was determined using culture media of cells seeded at a density of 2 × 10^5^ cell per well of 48-well plates in DMEM containing 10% FCS. After 24 h, cells were incubated with test substances or vehicles. For Western blot analyses of cell lysates (Figure 7B and 7D, lower blots) and cell culture media (Figure 7B, upper blot), 2 × 10^5^ cells were seeded into 6-well plates and treated using the protocol indicated under SiRNA transfections. Following incubation, cell culture media were obtained to analyse TIMP-1 release and cells were used to analyse TIMP-1, FAAH and β-actin from cell lysates. For analyses of TIMP-1 release indicated in Figure 7D, upper blot, cell culture media was used from cells seeded at a density of 5 × 10^5^ cells per well in 24-well plates and treated as described under SiRNA transfections. In transfection experiments for Western blot analyses presented in Figure 7, cells were retransfected in 500 μl of serum-free DMEM in 24-well plates (Figure 7D, upper blot) or 1 ml serum-free DMEM in 6-well plates (Figure 7B and 7D, lower blots) containing the same amounts of siRNA or non-silencing siRNA to provide constant transfection conditions.

In case of lysate analyses, cells were washed, harvested, lysed in solubilization buffer (50 mM HEPES, pH 7.4; 150 mM NaCl; 1 mM EDTA; 1% (v/v) Triton^®^ X-100; 10% (v/v) glycerol; 1 mM PMSF; 1 μg/ml leupeptin; 0.5 mM orthovanadate; and 10 μg/ml aprotinin), homogenized by sonication, and centrifuged at 10,000 g for 5 min. Supernatants were used for Western blot analysis.

Total protein in the cell lysates and the cell culture medium was measured using the Pierce^®^ bicinchoninic acid (BCA) protein assay kit (Thermo Scientific, Braunschweig, Germany). Denatured proteins were separated on a 10% sodium dodecyl sulfate-polyacrylamide gel (Applichem). Following transfer to nitrocellulose and blocking of the membranes with 5% milk powder, blots were probed with specific antibodies raised to TIMP-1 (Oncogene Research Products, San Diego, CA, USA), FAAH (Abcam, Cambridge, UK) or β-actin (Sigma). Membranes were probed with horseradish peroxidase-conjugated Fab-specific anti mouse IgG (New England BioLabs, Frankfurt, Germany) for detection of TIMP-1, FAAH and β-actin.

Densitometric analysis of band intensities was achieved by optical scanning and quantifying using the Quantity One 1-D Analysis Software (Bio-Rad Laboratories GmbH, Munich, Germany). Vehicle controls were defined as 100% for evaluation of changes in protein expression. To ensure that equal amounts of proteins of the cell culture media were loaded in the respective lanes and transferred to the membranes, proteins on Western blot membranes were stained with Ponceau Red (Carl Roth, Karlsruhe, Germany). To ascertain equal protein loading in Western blots of cell culture medium, a band with a size of ~ 65 kDa that appeared unregulated is shown as a loading control for protein analysis of cell culture medium. To ascertain equal protein loading in Western blots of cell lysates in Figure 7, membranes were probed with an antibody raised to β-actin. Densitometric values of TIMP-1 and FAAH obtained from analyses of cell lysates were normalized to those of Δ-actin.

### SiRNA transfections

Cells were transfected with small-interfering (si) RNA targeting TIMP-1 or FAAH (Qiagen) using RNAiFect^®^ as transfection reagent (Qiagen) or nonsilencing negative control RNA (Eurogentec, Köln, Germany). Target sequences were 5′-CAAGGCCCAACTAACAGTCAA-3′ for TIMP-1 siRNA and 5′-TCCCATCTTTCTTCCGGACAA-3′ for FAAH siRNA. A BLAST search revealed that the sequences selected did not show any homology to other known human genes. Transfections were performed according to the manufacturer's instructions.

For invasion assays, cells grown to confluence were transfected with siRNA or non-silencing siRNA as negative control with an equal ratio (w/v) of RNA to transfection reagent for 24 h in medium supplemented with 10% FCS. Subsequently, cells were trypsinised, centrifuged at 200 x g, resuspended to a final density of 5 × 10^5^ cells in 500 μl of serum-free DMEM per insert containing the same amounts of siRNA or non-silencing siRNA to provide constant transfection conditions, and seeded for invasion analysis as described above. For Western blot analyses indicated in Figure 7, cells received the second transfection without trypsinization.

### LC-MS analyses

A549 and H460 cells were seeded in Petri dishes with a 10 cm diameter at a density of 2 × 10^6^ cells per Petri dish and grown at 37°C in DMEM supplemented with 10% FCS, 100 U/ml penicillin and 100 μg/ml streptomycin. After 24 h, cells were washed once with PBS and incubated in serum-free DMEM with vehicle, 10 μM AA-5HT or 10 μM URB597 for another 6 h. For 1 sample (vehicle or FAAH inhibitor), 6 Petri dishes were used. Subsequently, cells were harvested by scraping and cell pellets obtained after centrifugation (10 min, 2000 x g, 4°C) were frozen in liquid nitrogen and stored at −80°C prior to analysis. For determination of endocannabinoids, cell pellets were further resuspended in 1 ml of 20 mM Tris-HCl buffer (pH 6.8) spiked with 20 ng/ml of AEA-d_8_ and lysed using a Sonopols U-tip sonifier (Bandelin, Berlin, Germany) 3 times with a 15 × 5-s pulse at 75% power followed by a 60-s pause. The lysates were transferred to ice-cold screw-capped glass tubes. In parallel with standard solutions, samples were extracted and analysed as described recently [19]. Briefly, extracted samples (30 to 60 μl) were analysed on a Waters HPLC 2695 Separation Module using a Multospher 120 C18 column 125 × 2 mm, 5-μm particle size (CS-Chromatographie Service GmbH, Langerwehe, Germany) coupled with a guard column (20 × 2 mm, 5-μm particle size). Endocannabinoids and endocannabinoid-like substances were resolved using the mobile phase A (water containing 0.2% formic acid) and the mobile phase B (acetonitrile/2-propanol [60:40, v/v] containing 0.2% formic acid) at a flow rate of 0.15 ml/min. The elution scheme was as follows: linear increase of the mobile phase B from 65% to 80% in 10 min, isocratic at 80% of phase B in 3 min and linearly to 100% phase B in the following 6 min. Finally, the system was re-equilibrated at 35% phase A over 4 min. The HPLC column effluent was introduced into a Micromass Quatro Micro^TM^ API mass spectrometer (Waters, Milford, USA) and analyzed using electrospray ionization in the positive mode and a single ion monitoring (SIM) modus: *m/z* 300.8 for PEA, *m/z* 326.8 for OEA, *m/z* 348.8 for AEA, *m/z* 379.8 for 2-AG and *m/z* 356.8 for the internal standard (AEA-d_8_). The mass spectrometer and source parameters were set up as follows: capillary voltage 3.5 kV; cone voltage 20 and 24 V for AEA/AEA-d_8_/2-AG and PEA/OEA, respectively; source temperature 120°C; desolvation temperature 350°C; flow rate of desolvation gas 700 l/h. Dwell and delay times were 0.05 and 0.1 s, respectively. All instrument parameters for the monitored analytes were tuned by injecting standard solutions at a concentration of 100 ng/ml at 10 μl/min flow rate by a syringe pump. The data were acquired using MassLynx software version 4.1 (Micromass Ltd., Manchester, UK). Upon quantitation the signals obtained for each analyte was normalized to the amount of internal standard observed in the corresponding sample. No effect of additives (AA-5HT or URB597) was observed during preparation of calibration curves for each of the standards used. Finally, an aliquot of each lysate (10 μl) was used for quantification of total protein using the Pierce^®^ BCA protein assay kit (Thermo Scientific).

### Mouse model of tumor metastasis

Female athymic nude mice (NMRI-nu/nu) were given injections of A549 cells (1 × 10^6^ cells in 100 μl PBS per 10 g body weight) through the lateral tail vein and, after 24 h, were treated intraperitoneally with the respective test substance or vehicle. Test substances or its vehicles were administered every 72 h for 28 days. Total lungs of mice sacrificed one day thereafter were evaluated for metastatic nodules. To contrast lung nodules, lungs were fixed in Bouin's fluid (saturated picrinic acid, formaldehyde, glacial acetic acid, 15:5:1 [v/v/v]), and metastatic nodules were scored under a stereoscopic microscope. For histopathological examination, lung samples were fixed in 4% (v/v) formaline. Paraffin sections were stained with hematoxylin and eosin to visualize metastatic foci. Experiments were conducted in accordance with the policies of the local Animal Ethics Committee.

### Induction of A549 xenografts in nude mice

Tumors were induced in female nude mice (NMRI-nu/nu) by subcutaneous inoculation of 1 × 10^7^ A549 cells into the the right dorsal flank. Animals were injected intraperitoneally every 72 h with test substances or its vehicles for 28 days. The treatment was started 7 days after tumor induction. Tumor volume was calculated as (4π/3) x (width/2)^2^ x (length/2). Experiments were conducted in accordance with the policies of local Animal Ethics Committee.

### Statistics

Comparisons between 2 groups were performed with Student's t test. Comparisons among more than 2 groups were carried out with one-way ANOVA plus post hoc Bonferroni or Dunnett test. All statistical analyses were undertaken using GraphPad Prism 5.04 (GraphPad Software, Inc., San Diego, USA).

## Abbreviations

AA-5HT: arachidonoyl serotonin
AEA: N-arachidono-ylethanolamine
2-AG: 2-arachidonoylglycerol
AM-251: N-(Piperidin-1-yl)-5-(4-iodophenyl)-1-(2,4-dichlorophenyl)-4-methyl-1H-pyrazole-3-carboxamide
AM-630: {6-Iodo-2-methyl-1-[2-(4-morpholinyl)ethyl]-1H-indol-3-yl}(4-methoxyphenyl)methanone
CB_1_: cannabinoid receptor 1
CB_2_: cannabinoid receptor 2
FAAH: fatty acid amide hydrolase
MAGL: monoacylglycerol lipase
NSCLC: non-small cell lung cancer
OEA: N-oleoylethanolamine
PEA: N-palmitoylethanolamine
TRPV1: transient receptor potential vanilloid 1
URB597: [3-(3-carbamoylphenyl)phenyl] N-cyclohexylcarbamate
